# Exposure of Neonatal Rats to Parathion Elicits Sex-Selective Impairment of Acetylcholine Systems in Brain Regions during Adolescence and Adulthood

**DOI:** 10.1289/ehp.11451

**Published:** 2008-05-19

**Authors:** Theodore A. Slotkin, Bethany E. Bodwell, Ian T. Ryde, Edward D. Levin, Frederic J. Seidler

**Affiliations:** 1 Department of Pharmacology and Cancer Biology; 2 Department of Psychiatry and Behavioral Sciences, Duke University Medical Center, Durham, North Carolina USA

**Keywords:** acetylcholine, brain development, organophosphate insecticides, parathion

## Abstract

**Background:**

Organophosphates elicit developmental neurotoxicity through multiple mechanisms other than their shared property as cholinesterase inhibitors. Accordingly, these agents may differ in their effects on specific brain circuits.

**Objectives:**

We gave parathion to neonatal rats [postnatal days (PNDs) 1–4], at daily doses of 0.1 or 0.2 mg/kg, spanning the threshold for barely detectable cholinesterase inhibition and systemic effects.

**Methods:**

We assessed neurochemical indices related to the function of acetylcholine (ACh) synapses (choline acetyltransferase, presynaptic high-affinity choline transporter, nicotinic cholinergic receptors) in brain regions comprising all the major ACh projections, with determinations carried out from adolescence to adulthood (PNDs 30, 60, and 100).

**Results:**

Parathion exposure elicited lasting alterations in ACh markers in the frontal/parietal cortex, temporal/occipital cortex, midbrain, hippocampus, and striatum. In cerebrocortical areas, midbrain, and hippocampus, effects in males were generally greater than in females, whereas in the striatum, females were targeted preferentially. Superimposed on this general pattern, the cerebrocortical effects showed a nonmonotonic dose–response relationship, with regression of the defects at the higher parathion dose; this relationship has been seen also after comparable treatments with chlorpyrifos and diazinon and likely represents the involvement of cholinesterase-related actions that mask or offset the effects of lower doses.

**Conclusions:**

Neonatal exposure to parathion, at doses straddling the threshold for cholinesterase inhibition, compromises indices of ACh synaptic function in adolescence and adulthood. Differences between the effects of parathion compared with chlorpyrifos or diazinon and the non-monotonic dose–effect relationships reinforce the conclusion that various organophosphates diverge in their effects on neurodevelopment, unrelated to their anticholinesterase actions.

Organophosphate (OP) pesticides damage the developing mammalian brain through multiple mechanisms that disrupt neural cell replication and differentiation, culminating in adverse effects on behavioral performance (Slotkin 1999, 2004, 2005). Recent studies confirm that even exposures that are not obviously symptomatic at or below the threshold for inhibition of cholinesterase, the standard biomarker for OP exposure and risk assessment, result in neurobehavioral abnormalities in animals (Slotkin 1999, 2004, 2005) as well as in children (Eskenazi et al. 2007; Landrigan 2001; Rauh et al. 2006; Young et al. 2005). Neural pathways involving acetylcholine (ACh) appear to be among those most sensitive to developmental disruption by OPs, partly because these agents specifically direct differentiation away from the ACh phenotype (Jameson et al. 2006; Slotkin et al. 2001, 2007a) and also because ACh neurons appear to be more vulnerable to toxic damage (Slotkin 1999, 2004, 2005). In turn, deficiencies of ACh function contribute directly to the emergence of cognitive impairment resulting from early-life exposure to OPs (Chanda and Pope 1996; Jett et al. 2001; Levin et al. 2001).

By far, chlorpyrifos is the OP that has been the most studied for developmental neurotoxicity (Slotkin 1999, 2004, 2005). However, because the adverse effects of these agents do not depend solely on their shared property of cholinesterase inhibition, the outcomes from exposures to other members of this pesticide class could differ; we conducted a series of studies demonstrating disparities in the initial neurotoxic injury and eventual synaptic and behavioral dysfunction between chlorpyrifos and diazinon (Jameson et al. 2007; Roegge et al. 2008; Slotkin et al. 2006a, 2006b, 2007c, 2008a, 2008b, 2008c; Slotkin and Seidler 2007a; Timofeeva et al. 2008). In the present study, we expanded our focus to include parathion, an OP that is far more systemically toxic to the neonate than either chlorpyrifos or diazinon (Kacham et al. 2006; Karanth and Pope 2003; Liu et al. 1999; Slotkin et al. 2006a). At its maximum tolerated dose, parathion produces less initial damage in the neonatal brain than does chlorpyrifos, largely because the maximum tolerated dose is so much lower due to higher systemic toxicity (Slotkin et al. 2006a). Nevertheless, there are substantial differences between the two agents in their impact on muscarinic ACh receptor expression in the neonate (Guo-Ross et al. 2007; Liu et al. 1999), suggesting that they also diverge in their ultimate effects on ACh neurotransmission. In the present study, we evaluated the long-term impact of neonatal parathion exposure on parameters of ACh synaptic function in adolescence, young adulthood, and full adulthood, focusing on doses spanning the threshold for signs of systemic toxicity: 0.1 mg/kg, a nonsymptomatic dose that produces barely detectable (10%) cholinesterase inhibition (Slotkin et al. 2006b), and 0.2 mg/kg, just over the threshold for the first signs of systemic toxicity (Slotkin et al. 2006a). Treatment was given during the immediate postnatal period [postnatal days (PNDs) 1–4], a stage at which we previously found high sensitivity of ACh systems to disruption by chlorpyrifos or diazinon (Roegge et al. 2008; Slotkin 1999, 2004, 2005; Slotkin et al. 2008a, 2008b; Timofeeva et al. 2008). Studies were conducted in various brain regions that comprise most of the major ACh projections: frontal/parietal cortex, temporal/occipital cortex, hippocampus, striatum, and midbrain.

We assessed three markers related to ACh function: activity of choline acetyltransferase (ChAT), cell membrane binding of hemicholinium-3 (HC3) to the presynaptic high-affinity choline transporter, and the concentration of α4β2 nicotinic acetylcholine receptors (nAChRs). ChAT is the enzyme that synthesizes ACh; because it is a constitutive component of ACh nerve terminals, its activity provides an index of the development of ACh projections (Dam et al. 1999; Happe and Murrin 1992; Monnet-Tschudi et al. 2000; Qiao et al. 2003; Richardson and Chambers 2005; Slotkin et al. 2001). Although HC3 binding to the choline transporter is also a constituent of ACh nerve terminals, its expression is directly responsive to neuronal activity (Klemm and Kuhar 1979; Simon et al. 1976), so that comparative effects on HC3 binding and ChAT enable the characterization of both the development of innervation and presynaptic activity. These two markers have been used to evaluate the effects of chlorpyrifos on ACh systems in adult rats (Liu and Pope 1996, 1998) and to characterize the immediate and delayed effects of postnatal chlorpyrifos or diazinon exposure (Dam et al. 1999; Rhodes et al. 2004; Richardson and Chambers 2005; Slotkin et al. 2001, 2006a, 2008a). Last, the α4β2 nAChR is a key player in the ability of ACh systems to release other neurotransmitters involved in reward, cognition, and mood (Buisson and Bertrand 2001, 2002; Dani and De Biasi 2001; Fenster et al. 1999; Quick and Lester 2002) and is also the most abundant nAChR subtype in the mammalian brain (Flores et al. 1992; Happe et al. 1994; Whiting and Lindstrom 1987, 1988).

## Materials and Methods

### Animal treatments

All experiments were carried out humanely and with regard for alleviation of suffering, with protocols approved by the Institutional Animal Care and Use Committee and in accordance with all federal and state guidelines. Timed-pregnant Sprague–Dawley rats were housed in breeding cages, with a 12-hr light–dark cycle and free access to food and water. On the day after birth, all pups were randomized and redistributed to the dams with a litter size of 10 (five males, five females) to maintain a standard nutritional status. Because of its poor water solubility, parathion was dissolved in dimethyl sulfoxide (DMSO) to provide consistent absorption (Slotkin et al. 2006a, 2006b; Whitney et al. 1995) and was injected subcutaneously in a volume of 1 mL/kg once daily on PNDs 1–4; control animals received equivalent injections of the DMSO vehicle, which does not itself produce developmental neurotoxicity (Whitney et al. 1995). Doses of 0.1 and 0.2 mg/kg/day were chosen because they straddle the threshold for barely detectable cholinesterase inhibition and the first signs of impaired viability (Slotkin et al. 2006a, 2006b). The low dose produces 5–10% inhibition without mortality, whereas the higher dose elicits 5–10% mortality. The PND1–4 regimen was chosen because it represents a peak period for sensitivity to the developmental neurotoxicity of chlorpyrifos (Slotkin 1999, 2004, 2005) and because the systemic toxicity and cholinesterase inhibition in response to parathion have already been characterized (Slotkin et al. 2006a, 2006b). Randomization of pup litter assignments within treatment groups was repeated at intervals of several days up until weaning, coordinated with weighing of the animals and changes of cage bedding. In addition, dams were rotated among litters to distribute any maternal caretaking differences randomly across litters and treatment groups. Offspring were weaned on PND21.

On PNDs 30, 60, and 100, one male and one female were selected from each litter of origin and were decapitated. The cerebellum (including flocculi) was removed, and the midbrain/brainstem was separated from the forebrain by a cut rostral to the thalamus. The striatum and hippocampus were then dissected from these larger divisions, and the midbrain and brainstem were divided from each other. The cerebral cortex was divided down the midline and then further sectioned into anterior and posterior regions (frontal/parietal cortex and temporal/occipital cortex, respectively). The cerebellum, which is sparse in ACh projections, was reserved for future studies. Tissues were frozen with liquid nitrogen and stored at −45°C.

### Assays

Tissues were thawed in 79 volumes of ice-cold 10 mM sodium–potassium phosphate buffer (pH 7.4) and homogenized with a Polytron (Brinkmann Instruments, Westbury, NY). Duplicate aliquots of the homogenate were assayed for ChAT using established procedures (Qiao et al. 2003, 2004). Each tube contained 50 μM [^14^C]acetyl-coenzyme A as a substrate, and activity was determined as the amount of labeled ACh produced relative to tissue protein (Smith et al. 1985).

For measurements of HC3 binding, the cell membrane fraction was prepared from an aliquot of the same tissue homogenate by sedimentation at 40,000 × *g* for 15 min. The pellet was resuspended and washed, and the resultant pellet was assayed with established procedures (Qiao et al. 2003, 2004), using a ligand concentration of 2 nM [^3^H]HC3 with or without 10 μM unlabeled HC3 to displace specific binding. Determinations of nAChR binding were carried out in another aliquot, each assay containing 1 nM [^3^H]cytisine with or without 10 μM nicotine to displace specific binding (Slotkin et al. 2008a). Binding was calculated relative to the membrane protein concentration.

### Data analysis

Data were compiled as means and standard errors. Because we evaluated multiple neurochemical variables that were all related to ACh synapses, the initial comparisons were conducted by a global analysis of variance (ANOVA) (data log-transformed because of heterogeneous variance among ages, regions, and measures) incorporating all the variables and measurements to avoid an increased probability of type 1 errors that might otherwise result from multiple tests of the same data set. Where we identified interactions of treatment with the other variables, data were then subdivided for lower-order ANOVAs to evaluate treatments that differed from the corresponding control. Where permitted by the interaction terms, individual groups that differed from controls in a given region at a given age were identified with Fisher’s protected least significant difference test. Significance was assumed at *p* < 0.05. For convenience, some of the results are presented as the percent change from control values, but statistical comparisons were conducted only on the original data. Although not shown here, the control values for each variable were quite similar to those published in our previous report (Slotkin et al. 2008a).

In evaluating the magnitude of the changes elicited by parathion administration, it is important to note that we used entire brain regions rather than specific nuclei, which means that even drastic effects on a specific population of neurons show up as smaller changes because of dilution with unaffected areas. Despite this limitation, we found statistically significant alterations for both treatment paradigms in multiple regions.

### Materials

Animals were obtained from Charles River (Raleigh, NC), and parathion was purchased from Chem Service (West Chester, PA). The radioisotopically labeled compounds [^14^C]acetyl-coenzyme A (specific activity 60 mCi/mmol, diluted with unlabeled compound to 6.7 mCi/mmol), [^3^H]HC3 (125 Ci/mmol), and [^3^H]cytisine (35 Ci/mmol) were obtained from PerkinElmer Life Sciences (Boston, MA). All other chemicals were purchased from Sigma Chemical Co. (St. Louis, MO).

## Results

Multivariate ANOVA examining all treatments, all brain regions, all ages, both sexes, and all three ACh synaptic measures identified a significant main treatment effect (*p* < 0.02) as well as interactions of treatment × sex (*p* < 0.03), treatment × region (*p* < 0.04), treatment × region × measure (*p* < 0.05), and treatment × sex × age × measure (*p* < 0.05). Because the chief interactions were with sex and region, we separated the values for males and females and examined the treatment effects and interactions within each region.

**Figure 1:**
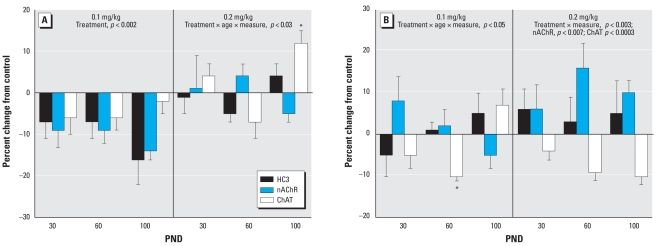
Effects of neonatal parathion exposure on development of ACh synaptic markers in the frontal/parietal cortex of (*A*) males and (*B*) females. Data represent means and SEs obtained from six males and six females in each treatment group at each age, presented as the percent change from control values. ANOVA across all treatments, ages and measures: for (*A*), treatment, *p* < 0.0005; treatment × age × measure, *p* < 0.03; and for (*B*) treatment × measure, *p* < 0.003; treatment × age × measure, *p* < 0.05. Lower-order ANOVAs for each dose are shown within the panel. Where there was a treatment interaction with both age and measure in the latter test, asterisks denote individual values that differ from the corresponding control; otherwise, only the ages or measures showing main treatment effects are listed.

In the frontal/parietal cortex, the low dose of parathion elicited a significant overall decrement in the three ACh markers in males (Figure 1A). Raising the dose to 0.2 mg/kg resulted in an attenuation of the effect; the only significant change was an increase in ChAT at PND100. In females, neonatal exposure to 0.1 mg/kg parathion failed to elicit the global decrease in ACh markers that had been seen in males (Figure 1B). At the higher dose, females showed a deficit in ChAT but an increase in nAChR binding; the latter effect was not seen in males at either dose.

**Figure 2:**
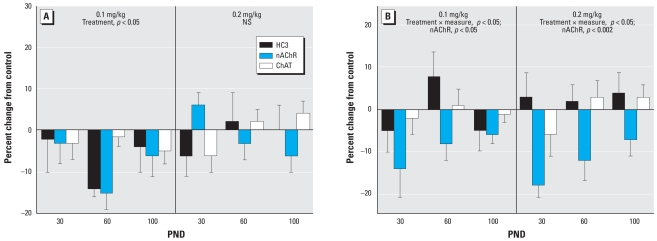
Effects of neonatal parathion exposure on development of ACh synaptic markers in the temporal/occipital cortex of (*A*) males and (*B*) females. NS, not significant. Data represent means and SEs obtained from six males and six females in each treatment group at each age, presented as the percent change from control values. ANOVA across all treatments, ages and measures: for (*A*), treatment × age × measure, *p* < 0.05; and for (*B*) treatment × measure, *p* < 0.02. Lower-order ANOVAs for each dose are shown within the panel. Because treatment did not interact with both age and measure, only the ages or measures showing main treatment effects are listed.

In males, the more caudal cerebrocortical regions (temporal/occipital cortex) again showed an overall decrease across the ACh markers (Figure 2A). As in the frontal/parietal cortex, increasing the neonatal exposure to 0.2 mg/kg resulted in a smaller change, connoting a nonmonotonic dose–effect relationship. In the temporal/occipital cortex, females showed significant effects at either dose of parathion, characterized by a decrement in nAChR binding (Figure 2B).

**Figure 3:**
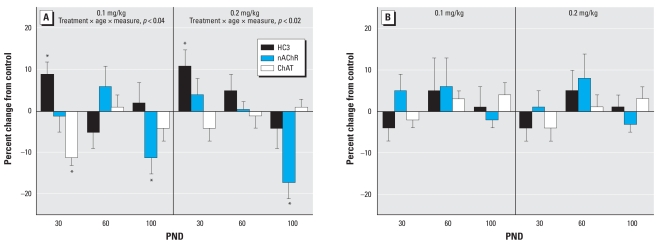
Effects of neonatal parathion exposure on development of ACh synaptic markers in the hippocampus of (*A*) males and (*B*) females. Data represent means and SEs obtained from six males and six females in each treatment group at each age, presented as the percent change from control values. ANOVA across all treatments, ages, and measures: for (*A*), treatment × age × measure, *p* < 0.03; and for (*B*), not significant. Lower-order ANOVAs for each dose are shown within the panel. Because treatment interacted with both age and measure in males, asterisks denote individual values that differ from the corresponding control.

**Figure 4:**
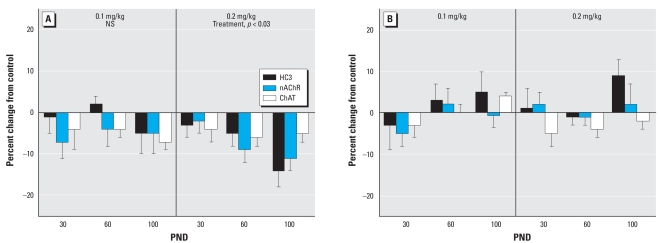
Effects of neonatal parathion exposure on development of ACh synaptic markers in the midbrain of (*A*) males and (*B*) females. NS, not significant. Data represent means and SEs obtained from six males and six females in each treatment group at each age, presented as the percent change from control values. ANOVA across all treatments, ages and measures: for (*A*), treatment, *p* < 0.05; and for (*B*) NS. Lower-order ANOVAs for each dose are shown within the panels. Because treatment did not interact with other variables, only main treatment effects are listed.

Like the cerebrocortical regions, the hippocampus displayed greater effects of neonatal parathion exposure on ACh synaptic parameters in males than in females. At either dose, males showed an early increase in HC3 binding and a later decrement in nAChR binding (Figure 3A). ChAT was depressed significantly on PND30 at the low dose, whereas at the higher dose there was a smaller effect that did not achieve statistical significance. However, these two effects were not themselves statistically distinguishable from each other. Females showed no significant effects in the hippocampus at either dose of parathion (Figure 3B). The same sex selectivity was evident in the midbrain. In this region, exposure of male neonates to 0.1 mg/kg parathion had little or no effect on ACh markers, but raising the dose to 0.2 mg/kg elicited significant deficits (Figure 4A). However, no significant effects were seen in the midbrain of females (Figure 4B).

**Figure 5:**
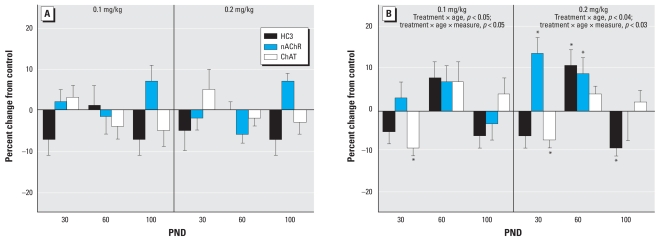
Effects of neonatal parathion exposure on development of ACh synaptic markers in the striatum of (*A*) males and (*B*) females. Data represent means and SEs obtained from six males and six females in each treatment group at each age, presented as the percent change from control values. ANOVA across all treatments, ages, and measures: for (*A*), not significant; and for (*B*) treatment × age × measure, *p* < 0.05. Lower-order ANOVAs for each dose are shown within the panel. Because treatment interacted with both age and measure in females, asterisks in (*B*) denote individual values that differ from the corresponding control. In addition, because of the treatment × age interaction, a lower-order test conducted for each age point identified main treatment effects on PND60 at each dose (*p* < 0.05 for both 0.1 and 0.2 mg/kg).

The effects in the striatum were distinct from those in the other regions. Males showed no significant net effects after neonatal parathion exposure (Figure 5A), whereas females showed significant changes at either dose (Figure 5B). At 0.1 mg/kg, there was a transient suppression of ChAT, and then a small, but significant overall elevation across all three parameters by PND60, regressing to normal values by full adulthood (PND100). At the higher dose, we found the same transient suppression of ChAT but also significant upregulation of nAChR binding. The higher dose also produced an elevation on PND60 and regression of values by PND100, except that the decrement in HC3 binding that was not significant at the lower dose now became significant at 0.2 mg/kg.

Neonatal parathion treatment did not significantly affect body weights in the animals studied here on PNDs 30, 60, and 100, nor were there any significant differences in brain region weights (data not shown). However, in the preweaning period, across the entire cohort of animals (a greater number than those used for the neurochemical studies presented here), we did find a significant growth effect (*p* < 0.005 for treatment, *p* < 0.003 for treatment × age), with the main effect confined to the group receiving 0.2 mg/kg (*p* < 0.0001) representing approximately a 5% difference from control values. We also evaluated large numbers of animals through 22 weeks postpartum, well beyond the period studied here, and found small, later-emerging deficits at either dose in females, again amounting to about 5% (data not shown).

## Discussion

There are two important conclusions from the current results. First, nonsymptomatic developmental exposures to parathion straddling the threshold for cholinesterase inhibition nevertheless produce lasting changes in ACh synaptic function. Second, although the effects of parathion bear resemblance to those of other OPs such as chlorpyrifos (Slotkin et al. 2001) or diazinon (Slotkin et al. 2008a), there are also notable differences. This reinforces the idea that the developmental neurotoxicity of OPs involves mechanisms other than their shared property of cholinesterase inhibition. Thus, although all three agents target ACh systems in the forebrain and midbrain areas comprising the major ACh projections, they differ in their regional targeting and sex selectivity. Although we observed effects in both sexes, parathion had much more widespread effects in males than in females, continuing a general pattern seen with the other OPs (Slotkin et al. 2001, 2008a). We observed sex differences for each region and compared the pattern of effects seen with parathion exposure to those of chlorpyrifos and diazinon.

In the two cerebrocortical areas as well as in the midbrain, parathion caused deficits in all ACh-related markers in males. In contrast, females showed effects in temporal/occipital cortex, accompanied by suppression of nAChRs, an effect that was not seen in males. Thus, there are two important considerations of this set of results, namely, the strong sex differences and the fact that many of the results show a nonmonotonic dose–effect relationship, with lesser effects of parathion at the higher dose. Sex differences in the developmental neurotoxicity of OPs are commonly observed with chlorpyrifos and diazinon (Aldridge et al. 2004, 2005; Dam et al. 2000; Levin et al. 2001, 2002; Moser et al. 1998; Ricceri et al. 2006; Roegge et al. 2008; Slotkin et al. 2001, 2002, 2006b, 2008a, 2008b; Slotkin and Seidler 2005, 2007b; Timofeeva et al. 2008) and reflect two contributing mechanisms. First, these agents appear to interfere with sexual differentiation of the brain, narrowing or eliminating many of the normal sex differences in behavioral and/or neurochemical parameters (Aldridge et al. 2005; Levin et al. 2001; Slotkin 1999, 2004, 2005). Second, even where the initial neurotoxicity might be equivalent in both sexes, the subsequent repair processes differ substantially, with females showing a greater general capacity to offset damage (Amateau and McCarthy 2002; Hilton et al. 2004; McEwen 2002; Nunez and McCarthy 2003; Slotkin et al. 2007b; Tanapat et al. 1999). The reduction in nAChR expression in females is thus likely to represent a component of the adaptive mechanisms required for reprogramming of synaptic circuits that may aid in restoring function. In any case, the adverse effects of nonsymptomatic neonatal parathion exposure on frontal cortex ACh circuits are likely to have important parallels in behavioral performance, as these pathways are critically involved in attention (Passetti et al. 2000).

A nonmonotonic dose–response relationship typically connotes the existence of multiple mechanisms of action, so that the net outcome is a superimposition of the various dose–effect curves, each of which occupies a different dose range. Here, we observed greater effects of 0.1 mg/kg parathion than 0.2 mg/kg for effects on the cerebrocortical regions in males, repeating a pattern noted earlier for chlorpyrifos and diazinon (Levin et al. 2002; Slotkin et al. 2008a; Timofeeva et al. 2008). The likely mechanism for the reduced effect at the higher dose is the greater degree of cholinesterase inhibition (Slotkin et al. 2006b). In the developing brain, ACh acts as a neurotrophic factor that promotes the survival and differentiation of its target cells (Hohmann 2003; Lauder and Schambra 1999; Picciotto and Zoli 2008), an effect that can be mimicked in part by dietary choline supplementation (Meck and Williams 1997; Montoya et al. 2000). Accordingly, a small degree of cholinesterase inhibition, too low to elicit systemic toxicity or other adverse effects of ACh hyperstimulation, might offset some of the adverse effects of parathion that are mediated through noncholinesterase mechanisms operating at lower exposures. In a recent study, Laviola et al. (2006) demonstrated conclusively that a carefully chosen, small dose of chlorpyrifos can accelerate some aspects of neurodevelopment, even while damaging other aspects.

Neonatal parathion exposure had a less notable effect in the hippocampus as opposed to the cerebrocortical regions, with a reduction in nAChRs in males as the only persistent effect. This stands in direct contrast to the major deficits in HC3 binding seen after comparable treatment with chlorpyrifos (Slotkin et al. 2001) or reductions in ChAT seen with diazinon (Slotkin et al. 2008a). Because ACh projections to the hippocampus provide major contributions to visuospatial memory performance, we clearly expect to see significant divergence in behavioral outcomes for the three different OPs. Indeed, we have already seen such disparities between chlorpyrifos and diazinon, including different patterns of sex selectivity (Aldridge et al. 2005; Levin et al. 2001; Roegge et al. 2008; Timofeeva et al. 2008), and studies are currently under way for parathion. Again, our specific finding here of deficient nAChR expression is likely to be involved in behavioral deficits, as suppression of the α4β2 subtype in the hippocampus impairs working memory performance (Pocivavsek et al. 2006).

In the midbrain, we again saw greater targeting of males than females. Although we found the same pattern for neonatal chlorpyrifos exposure (Slotkin et al. 2001), comparable treatment with diazinon does not seem to target these projections (Slotkin et al. 2008a). Unlike the situation in the cerebrocortical regions, the dose–effect relationship for parathion was monotonic, with substantially greater effects at the higher dose. This points out again that the net outcomes after neonatal OP exposure are dependent on multiple factors that may diverge in major ways depending upon the individual agent and the brain region, especially given that each region is at a different maturational stage during any specific window of exposure (Rodier 1988). Indeed, although we studied ACh projections in all the regions, each synaptic population originates from cells in different brain areas that undergo differentiation and synaptic outgrowth with widely varying timetables (Rodier 1988). The ACh innervation of the midbrain originates in the pedunculopontine and dorsolateral tegmental nuclei; that in the hippocampus comes from the medial septal nucleus and the nucleus of the diagonal band; the projections to the cerebral cortex derive from nuclei of the basal forebrain; and striatal ACh terminals primarily represent interstitial ACh interneurons. Finally, the striatum was the one region where the long-term disruption was greater in females than males, again continuing a regional distinction noted in our earlier work with the other OPs (Slotkin et al. 2001, 2008a). In the present study, parathion elicited nAChR upregulation, whereas there were deficits in HC3 indicative of impaired synaptic activity. This pattern is essentially identical to that seen after either neonatal chlorpyrifos or diazinon exposure (Slotkin et al. 2001, 2008a), so that we would not expect to see major differences among the three agents in behavioral outcomes related to this set of pathways. Impaired striatal ACh function is likely to affect a number of related learning tasks (Legault et al. 2006; Palencia and Ragozzino 2006).

## Conclusion

Our results in the present study reinforce the basic finding that OPs are developmental neurotoxicants at exposures below the threshold for any signs or symptoms of acute systemic toxicity, and at or below the threshold for cholinesterase inhibition. These agents elicit persistent effects on ACh systems critically involved in learning and memory functions. Although, as studied here, many of the effects of parathion resemble those seen in earlier work with chlorpyrifos or diazinon (Slotkin et al. 2001, 2008a), the OPs all differ in their regional targeting and sex selectivity, representing the outcomes of mechanisms that are unrelated to their shared property as cholinesterase inhibitors. In turn, these individual attributes likely contribute to differences in behavioral outcomes, buttressing the need to evaluate multiple behavioral end points in both males and females with each agent. Further, the nonmonotonic relationship seen repeatedly for effects of OPs on both neurochemical and behavioral parameters (Levin et al. 2002; Qiao et al. 2002; Slotkin et al. 2008a; Timofeeva et al. 2008) points out the need to pursue studies at even lower exposures. Certainly, these results indicate the inadequacy of an approach that focuses solely on exposures at or above the maximum tolerated dose; given the multiple mechanisms underlying the developmental neurotoxicity of OPs, neurobehavioral damage may be revealed at lower doses that are devoid of countervailing secondary effects. Finally, although OPs in general target developing ACh pathways, the differences in outcomes after neonatal exposure to parathion compared with chlorpyrifos or diazinon indicate the need to consider each agent individually for its propensity to elicit neurodevelopmental damage, and point to the potential to design safer members of this class of pesticides.
